# Calibration Method of an Ultrasonic System for Temperature Measurement

**DOI:** 10.1371/journal.pone.0165335

**Published:** 2016-10-27

**Authors:** Chao Zhou, Yueke Wang, Chunjie Qiao, Weihua Dai

**Affiliations:** College of Mechatronics Engineering and Automation, National University of Defense Technology, Changsha, Hunan Province, China; Nanjing Normal University, CHINA

## Abstract

System calibration is fundamental to the overall accuracy of the ultrasonic temperature measurement, and it is basically involved in accurately measuring the path length and the system latency of the ultrasonic system. This paper proposes a method of high accuracy system calibration. By estimating the time delay between the transmitted signal and the received signal at several different temperatures, the calibration equations are constructed, and the calibrated results are determined with the use of the least squares algorithm. The formulas are deduced for calculating the calibration uncertainties, and the possible influential factors are analyzed. The experimental results in distilled water show that the calibrated path length and system latency can achieve uncertainties of 0.058 mm and 0.038 μs, respectively, and the temperature accuracy is significantly improved by using the calibrated results. The temperature error remains within ±0.04°C consistently, and the percentage error is less than 0.15%.

## Introduction

Ultrasonic temperature measurement, as a non-intrusive technique, plays a crucial role in plenty of industrial processes due to its strong environmental adaptability [1, 2], high reliability [3], low cost [4] and wide measuring range [5]. The basic principle is that the ultrasound velocity in any medium is generally a function of temperature. In most liquids, the function is linear; in a gaseous environment, the velocity is directly proportional to the square root of the temperature; and in solid mediums, the velocity generally decreases with the increasing of the temperature [6]. Thus, if the ultrasound velocity is measured, the temperature can be determined. In our previous study [7], we proposed a promising method for highly accurate ultrasonic temperature measurement. Two transducers, mounted face to face, act as the transmitter and the receiver of the ultrasound signals, respectively, and the distance between them is fixed. By estimating the time delay (TD) between the transmitted signal and the received signal, the ultrasound velocity is given, and the temperature can be determined.

However, the estimated TD contains not only the desired time of flight of sound, but also a system latency which includes the time taken by the transmitter to produce the sound, the time taken by the receiver to produce the electrical signal and the time taken by the conditioning circuit [8]. The system latency is an intrinsic hardware constant that need to be estimated in advance. Moreover, the path length of sound is generally not the transducers’ geometrical distance, but the travelling sound path length which serves as the acoustic center-to-center distance [5]. The exact path length is not available from direct distance measurement. Therefore, to accurately estimate the path length and the system latency, calibration work of the ultrasonic system should be performed, which is the basis of high accuracy ultrasonic temperature measurement.

The calibration methods of path length usually depend on measurement of the acoustic centers of the transducers. Based on the definition that the acoustic center is the position of the point from which the sound pressure varies inversely as distance [9, 10], most results presented in the literature have been determined from deviations of the amplitude of the sound pressure [11]. Cox attempted to measure the acoustic centers of various transducers using a dismantled lathe bed for positioning the scanning microphone [12]. The comparisons of acoustic centers among several European laboratories were summarized by Rasmussen and Olsen [13]. Juhl [14] measured the acoustic centers by using the boundary value technique and assuming a parabolic movement of the diaphragms. Barrera-Figueroa et al. [15] presented an experimental procedure to determine the position of the acoustic centers of the microphones. Shaw and Hodnett [16] described some typical issues related to calibration and measurement of therapeutic medical ultrasonic equipment. A theoretical model for the transfer characteristics of a hydrophone has been developed, which can help to calculate the system latency [17]. However, the methods mentioned above mainly focused on calibrating the characteristics of the transducers, rather than the ultrasonic system directly. The calibration procedure is very complex, and it is difficult to achieve high calibration performance.

This paper proposes a promising calibration method to determine the path length and the system latency of an ultrasonic system for temperature measurement. Calibration equations on the path length and the system latency are presented by estimating the TD between the transmitted signal and received signal at several different temperatures, and the calibrated results are determined simultaneously based on the least squares algorithm. Distilled water is employed as the medium to perform the experiment keeping in a stable temperature environment with an accuracy of 0.01°C. To achieve highly accurate TD estimation, a continuous wave modulated by maximum length sequence is adopted, and a hybrid method is employed by incorporating both cross-correlation and phase shift. According to the basic calibration principle, the given TD uncertainty and the thermometer uncertainty, the formulas for calculating the path length uncertainty and the system latency uncertainty are presented, and the uncertainty propagation coefficients are also deduced. To validate the effectiveness of the proposed method, the calibrated results are used in ultrasonic temperature measurement, which shows the temperature accuracy is significantly improved.

## Problem formulation

**Fig 1:**
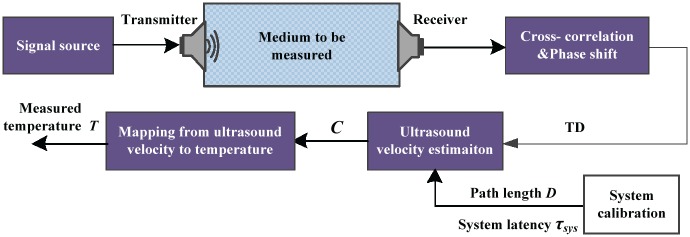
Basic principle of ultrasonic temperature measurement.

In Ref. [7], the authors proposed a promising method for ultrasonic temperature measurement. and the problem of system calibration was also mentioned. In this section, a brief overview is given. The graphic abstract of temperature measurement is shown in Fig 1. If equation *C* = *f*(*T*) represents the inner dependence between the temperature (*T*) and the ultrasound velocity (*C*), then *T* can be determined when *C* is measured

$$\begin{array}{c} T=f^{-1}\left(C\right), \end{array}$$ (1)

where *f*^−1^() denotes the mapping relationship from the ultrasound velocity to the temperature. Signal source drives the transmitter to produce sound wave, which is captured by the receiver after propagation in the medium. A hybrid technique is adopted to estimate the TD between the transmitted signal and received signal by incorporating both cross-correlation and phase shift. Combining the estimated TD and the calibrated results of the ultrasonic system, the ultrasound velocity of the medium can be expressed as following:

$$\begin{array}{c} C=\frac{D}{\text{TD}-\tau_{sys}}, \end{array}$$ (2)

where *D* and *τ*_*sys*_ represent the path length and the system latency, respectively. The temperature to be measured is determined by

$$\begin{array}{c} T=f^{-1}\left(\frac{D}{\text{TD}-\tau_{sys}}\right). \end{array}$$ (3)

Accurate temperature is achievable on the basis of estimating TD accurately. And the inaccurate representation of *D* and *τ*_*sys*_ should be carefully considered. This paper is to calibrate the path length *D* and the system latency *τ*_*sys*_ exactly.

## Calibration of ultrasonic system

### Calibration method

TD estimation between the transmitted signal and the received signal is performed at *M* different known temperatures. The calibration equations on *D* and *τ*_*sys*_ are presented as following:

$$\begin{array}{c} \left\{ \begin{array}{c} TD_{1}=\frac{D}{f(T_{1})}+\tau_{sys}+e_{1}\\ TD_{2}=\frac{D}{f(T_{2})}+\tau_{sys}+e_{2}\\ \cdots \\ TD_{M}=\frac{D}{f(T_{M})}+\tau_{sys}+e_{M} \end{array},\right. \end{array}$$ (4)

where *TD*_*m*_ denotes the estimated time delay of the *m*-th temperature, and *e*_*m*_ is the corresponding estimation error. *f*(*T*_*m*_) denotes the ultrasound velocity related to the temperature *T*_*m*_, which is measured in advance. By using the least squares which is an optimal algorithm of parameter estimation for linear models [18], the calibrated path length ($\hat{D}$) and system latency (${\hat{\tau }}_{sys}$) can be deduced by

$$\begin{array}{c} {}[\begin{array}{c} \hat{D}\\ {\hat{\tau }}_{sys} \end{array}]={\left(A^{T}A\right)}^{-1}A^{T}X, \end{array}$$ (5)

where

$$\begin{array}{c} A=[\begin{array}{cc} 1/f(T_{1}) & 1\\ 1/f(T_{2}) & 1\\ \vdots & \vdots \\ 1/f(T_{M}) & 1 \end{array}],X=[\begin{array}{c} TD_{1}\\ TD_{2}\\ \vdots \\ TD_{M} \end{array}]. \end{array}$$ (6)

To ensure that Eq (5) can be deduced from Eq (4), the matrix **A** should has a full column rank, which requires *f*(*T*) to be a monotone function as *T* ranges from *T*_1_ to *T*_*M*_. And accurate system calibration is achievable on the basis of estimating time delay and temperature accurately.

### Experimental setup

**Fig 2:**
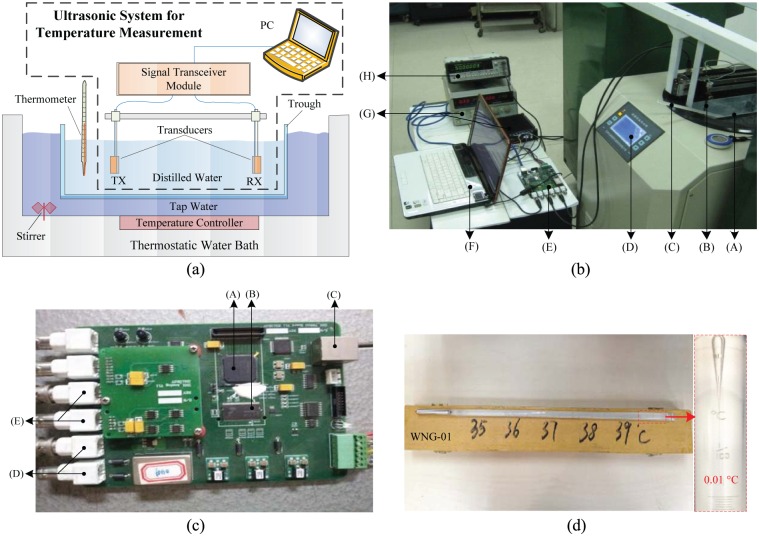
The experimental setup. (a) Schematic of the experimental system. (b) Photograph of the actual system. (A) is the trough with distilled water, (B) is one of the transducers, (C) is the mercuric thermometer, (D) is the thermostatic water bath, (E) is the signal transceiver module, (F) is the PC, (G) is the power supply and (H) is the frequency meter. (c) Photograph of the signal transceiver module. (A) is the FPGA, (B) is the DDR memory, (C) is the Ethernet port, (D) are the transmitting channels and (E) are the receiving channels. (d) The Photograph of one of the adopted mercuric thermometers, which ranges from 35°C ∼ 39°C.

The experimental setup is shown in Fig 2. It is a typical ultrasonic system for temperature measurement within the dashed box that consists of two transducers, a signal transceiver module and a PC. The transmitter and the receiver both use Vico’s WK-21B acoustic piezoelectric transducers, which work at 1 MHz and have an operating temperature within 5°C ∼ 110°C. The transducers are fixed on a steel framework face to face. The primary functions of the transceiver module, shown in Fig 2(c), include signal storage, signal transmission and signal receiving. The FPGA (XILINX XC6SLX45) works as a core function jointed with a 100M-Base PHY (LXT971ALE), an ADC (ADS2806Y), a DAC (DAC2932PFB), dual DDR (MT46V64M8TG) and other external ports. The transmitted signal, generated by the PC, is downloaded through the Ethernet to the DDR. System starts when the PC sends a trigger command. The DAC converts the digital signal from DDR into an analog signal to drive the transmitter. The ADC, with a sampling rate at 10 Msps, works synchronously with the DAC, and the sampled signal is fed back to the DDR and uploaded to the PC for further processing.

**Fig 3:**
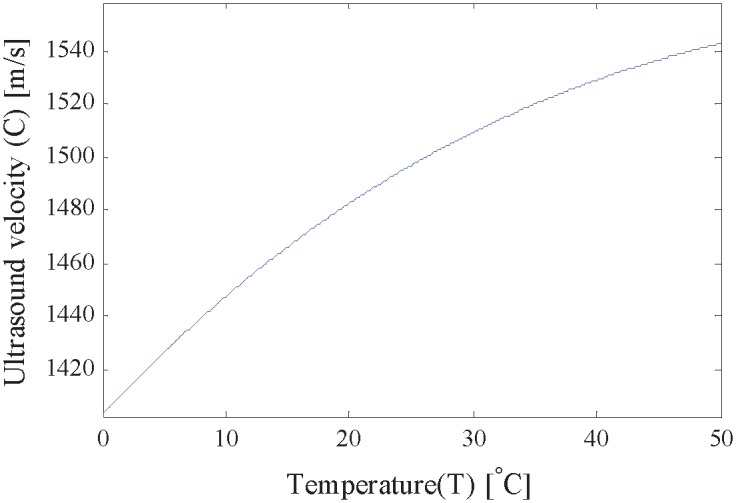
The function that ultrasound velocity versus temperature in distilled water.

Distilled water is selected as the medium to carry out the system calibration from two aspects of concern. First, it is widely accepted that the mapping function between the ultrasound velocity and the temperature in distilled water is sufficiently accurate. Additionally, the temperature of liquid such as distilled water has potential to be accurately measured by a mercuric thermometer, and the changes of temperature can be easily controlled. In 1972, Del Grosso first provided the ultrasound velocity equation of distilled water [19], and it was approximated by a polynomial [20]:

$$\begin{array}{c} C=k_{0}+k_{1}\cdot T+k_{2}\cdot T^{2}+k_{3}\cdot T^{3}+k_{4}\cdot T^{4}+k_{5}\cdot T^{5}, \end{array}$$ (7)

where *T* is the temperature in degrees Celsius, *k*_0_ = 0.140238744 × 10^4^, *k*_1_ = 5.03835027, *k*_2_ = −0.581142290 × 10^−1^, *k*_3_ = 0.334558776 × 10^−3^, *k*_4_ = −0.14815004 × 10^−5^, and *k*_5_ = 0.316081885 × 10^−8^. As shown in Fig 3, it is one to one relationship between *C* and *T*, which is a basic requirement of our proposed calibration method.

The two transducers are fully immersed into the distilled water, and the temperature is measured using a mercuric thermometer, manufactured by Jing Chuang Instrument (model WNG-01) with a typical accuracy of 0.01°C. A thermostatic water bath (BILOW-GDW-2011B) is used to monitor and control the temperature of tap water, which exchanges heat with the distilled water. Highly constant and accurate temperature of the distilled water is achievable on the basis of following techniques: (1) The ultrasound wave is not transmitted until the temperature stays at the target point for 10 minutes, which contributes to constructing a stable and uniform temperature environment. (2) By using a transmitted signal that consists of 40 sets of continuous wave, 40 TD estimations are completed in less than a second. In such a short period, the temperature of the distilled water is nearly constant. (3) At the moment of signal transmitting, the thermometer readings are recorded as soon as possible to ensure the accuracy of the temperature. Additionally, air conditioning in the laboratory is also turned on and set at the same temperature to reduce the temperature difference between the air and the distilled water.

At each temperature, an ultrasound wave is transmitted by the transmitter, and received by the receiver after propagation in the distilled water. To achieve highly accurate TD estimation, a continuous wave modulated by maximum length sequence is adopted for echo suppression to increase the available length of the received signal, and a hybrid technique incorporating both cross-correlation and phase shift is employed. Cross-correlation initially, based on the transmitted and received signals, produces a rough TD to eliminate the phase ambiguity, and then phase shift refines this rough value to estimate TD with higher accuracy [7].

## Experimental results and discussion

### Experimental results

We carried out system calibration at 7 different temperatures (*M* = 7) ranging from 18°C to 42°C, and TD estimation was repeated 40 times at each temperature. The average of estimated TD, summarized in Table 1, decreases with the increasing of the temperature and has a standard deviation of less than 0.3 ns. Based on Eqs (5) and (6), the path length and the system latency are determined as *D* = 185.2265 mm and *τ*_*sys*_ = 9.0171 μs, respectively.

**Table 1:** The calibrated results.

*T*(°C)	*C*(m/s)	*TD* (μs)		*D*	*τ*_*sys*_
		Average	Std		
18.073	1476.2865	134.4842	2.4 × 10^−5^	185.2265 mm	9.0171 μs
21.980	1488.2773	133.4747	3.4 × 10^−5^		
25.970	1499.2621	132.5615	2.4 × 10^−4^		
29.912	1508.9417	131.7711	4.2 × 10^−5^		
34.994	1519.8150	130.8949	1.5 × 10^−4^		
38.083	1525.5945	130.4241	7.5 × 10^−5^		
41.957	1532.0084	129.9231	1.4 × 10^−4^		

### Uncertainty analysis

Uncertainty analysis plays a crucial role in evaluation of the calibration performance, and it can help to find out which parameter has the biggest effect and which need only to be roughly given.

The calibrated path length and system latency, from Eq (5), can be formally expressed as

$$\begin{array}{c} \left\{ \begin{array}{c} \hat{D}=f_{D}(T_{1},\cdots ,T_{M},TD_{1},\cdots ,TD_{M})\\ {\hat{\tau }}_{sys}=f_{\tau }(T_{1},\cdots ,T_{M},TD_{1},\cdots ,TD_{M}) \end{array},\right. \end{array}$$ (8)

where *f*_*D*_() and *f*_*τ*_() represent the functions to determine the path length and the system latency, respectively. Because temperature measurement and TD estimation are independent, both path length uncertainty (*u*_*D*_) and system latency uncertainty (*u*_*τ*_) can be decomposed into two parts:

$$\begin{array}{c} \left\{ \begin{array}{c} u_{D}=\sqrt{u_{D1}^{2}+u_{D2}^{2}}\\ u_{\tau }=\sqrt{u_{\tau 1}^{2}+u_{\tau 2}^{2}} \end{array},\right. \end{array}$$ (9)

where *u*_*D*1_ and *u*_*D*2_ are the path length uncertainties introduced by temperature measurement and TD estimation, respectively. *u*_*τ*1_ and *u*_*τ*2_ are the system latency uncertainties introduced by temperature measurement and TD estimation, respectively. Moreover, the inner dependence of uncertainty propagation is strictly proportional, such that

$$\begin{array}{c} \left\{ \begin{array}{c} u_{D1}=\alpha_{T-D}\cdot u_{T},\;\;u_{D2}=\alpha_{\text{TD}-D}\cdot u_{\text{TD}}\\ u_{\tau 1}=\alpha_{T-\tau }\cdot u_{T},\;\;u_{\tau 2}=\alpha_{\text{TD}-\tau }\cdot u_{\text{TD}} \end{array},\right. \end{array}$$ (10)

where *u*_*T*_ denotes the temperature uncertainty, *α*_*T*−*D*_ and *α*_*T*−*τ*_ are the propagation coefficients to introduce *u*_*T*_ to *u*_*D*1_ and *u*_*τ*1_, respectively. *u*_*TD*_ denotes the TD uncertainty, *α*_*TD*−*D*_ and *α*_*TD*−*τ*_ are the propagation coefficients to introduce *u*_*TD*_ to *u*_*D*2_ and *u*_*τ*2_, respectively. These coefficients are determined by [21]

$$\begin{array}{c} \left\{ \begin{array}{cc} \alpha_{T-D}=\sqrt{\sum_{m=1}^{M}{\left(\frac{\partial f_{D}}{\partial T_{m}}\right)}^{2}}, & \alpha_{\text{TD}-D}=\sqrt{\sum_{m=1}^{M}{\left(\frac{\partial f_{D}}{\partial TD_{m}}\right)}^{2}}\\ \alpha_{T-\tau }=\sqrt{\sum_{m=1}^{M}{\left(\frac{\partial f_{\tau }}{\partial T_{m}}\right)}^{2}}, & \alpha_{\text{TD}-\tau }=\sqrt{\sum_{m=1}^{M}{\left(\frac{\partial f_{\tau }}{\partial TD_{m}}\right)}^{2}} \end{array}.\right. \end{array}$$ (11)

The detailed uncertainty of the experiment is shown in Table 2. The temperature uncertainty (*u*_*T*_) only depends on the accuracy of the mercuric thermometer. Generally, the temperature error satisfies a uniform distribution within ±0.01°C, so we have $u_{T}=0.01/\sqrt{3}\approx 0.006^{{}^{\circ}}\text{C}$ [21]. The TD uncertainty (*u*_*TD*_) is less than 0.3 ns, which depends on the parameters of the received signal, such as the signal-to-noise ratio, the carrier frequency and the signal length [22]. The uncertainties of the calibrated path length and system latency are close to 0.058 mm and 0.038 μs, respectively. And the temperature measurement has the biggest effect which is approximately 4 times bigger than the effect of TD estimation. To improve the calibration performance, three techniques are useful: (1) using a thermometer with higher accuracy; (2) improving the performance of TD estimation; (3) improving the calibration equations to reduce the uncertainty propagation coefficients.

**Table 2:** Uncertainty analysis of the calibrated results.

*u*_*T*_	*u*_TD_	Propagation coefficients	Path length uncertainty (mm)	System latency uncertainty (μs)
0.006°C	0.3 ns	*α*_*T*−*D*_: 9.813 mm/°C	*u*_*D*1_: 5.666 × 10^−2^ *u*_*D*2_: 1.360 × 10^−2^ *u*_*D*_: 5.826 × 10^−2^	*u*_*τ*__1_: 3.739 × 10^−2^ *u*_*τ*__2_: 9.024 × 10^−3^ *u*_*τ*_: 3.847 × 10^−2^
		*α*_*T*−*τ*_: 6.477 *μs*/°C		
		*α*_*TD*−*D*_: 0.045 mm/ns		
		*α*_*TD*−*τ*_: 30.080		

### Temperature measurement using calibrated results

To further validate the effectiveness of the proposed method, the calibrated results are used in ultrasonic temperature measurement. The experiment was performed at 13 different temperatures ranging from 18°C to 42°C, and the temperature, measured by the mercuric thermometer, is regarded as the reference true value. Another experiment was also carried out for comparison, where the system latency remained unchanged (*τ*_*sys*_ = 9.0171 μs), but the path length was measured by a slide caliper ruler with a typical accuracy of 0.02 mm. The measured path length is *D* = 184.80 mm.

**Fig 4:**
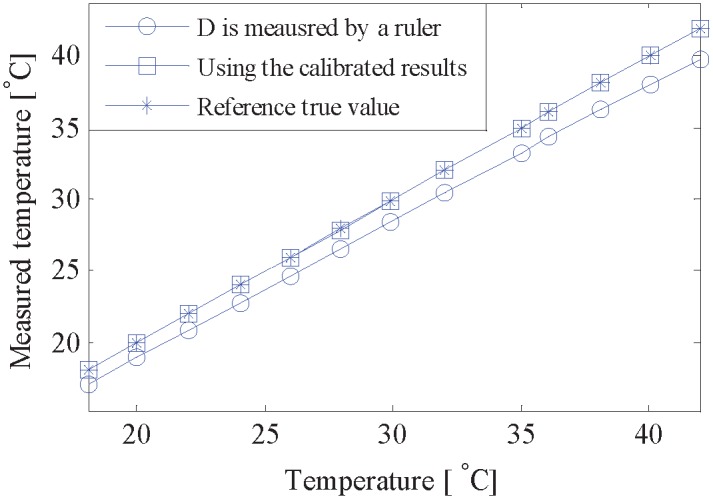
Results of measured ultrasonic temperature.

**Fig 5:**
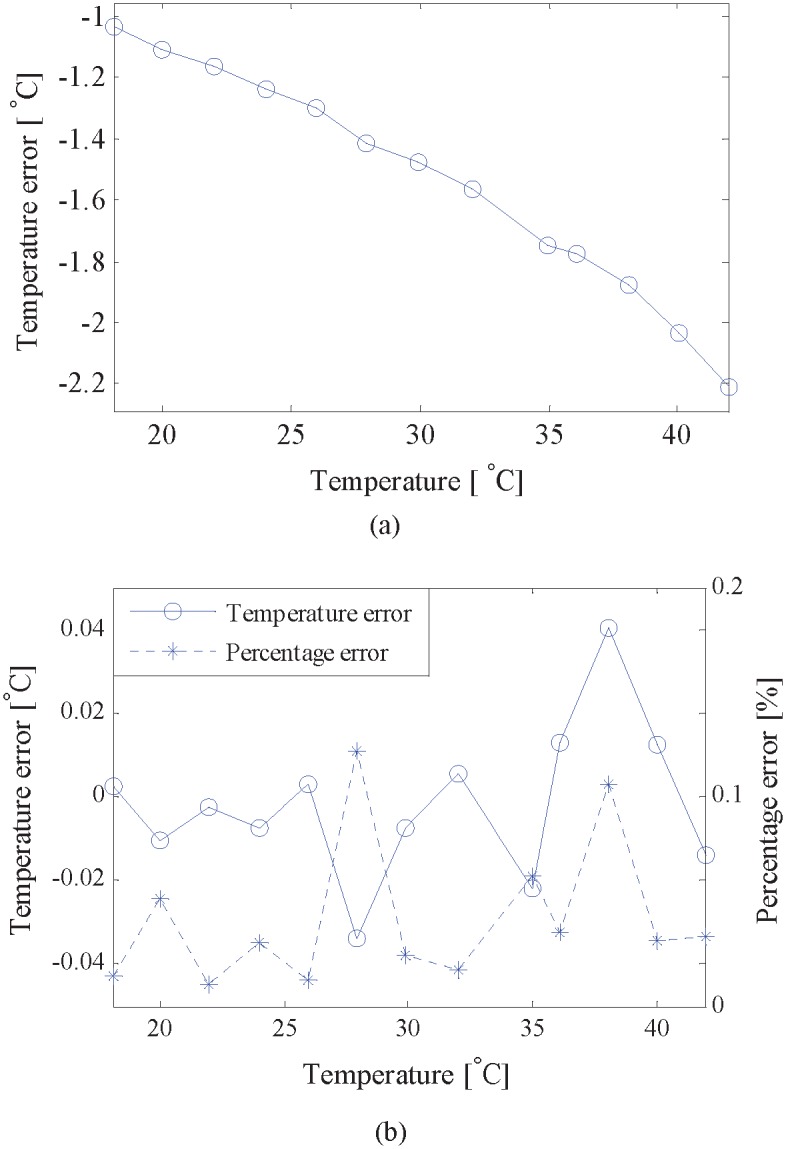
Temperature errors of ultrasonic measurement. (a) The path length is measured by a slide caliper ruler. (b) Using the calibrated results.

Fig 4 presents the results of measured temperature, where temperature in abscissa represents the reference true value determined by the thermometer. It is shown that the measured temperature, using calibrated results, is highly close to the reference true value. However, system error appears obviously when the path length is measured by a slide caliper ruler. And the system error, shown in Fig 5(a), increases with the rise of the temperature, which is up to 2.211°C when the temperature is 41.957°C. Fig 5(b) shows the temperature error by using the calibrated results, where the temperature accuracy is significantly improved. The temperature error consistently remains within ±0.04°C, and the percentage error is less than 0.15%.

## Conclusions

This paper presents a promising calibration method of an ultrasonic system for temperature measurement. And the following conclusions can be made:

- By estimating TD between the transmitted signal and received signal at several different temperatures, the calibration equations are presented, and the path length and the system latency are determined simultaneously based on the least squares algorithm;
- The formulas for calculating the calibration uncertainties are given, and the uncertainty propagation coefficients are also deduced, which plays a crucial role in finding out which parameter would have the biggest effect and which need only to be roughly considered;
- Both calibration experiment and validation experiment were performed in the distilled water in a stable temperature environment with an accuracy of 0.01°C;
- The proposed method calibrates the path length and the system latency with uncertainties of 0.058 mm and 0.038 μs, respectively. With the use of the calibrated results, the performance of temperature measurement is significantly improved. The temperature error consistently remains within ±0.04°C, and the percentage error is less than 0.15%.
